# Low Energy Consumption Compressed Spectrum Sensing Based on Channel Energy Reconstruction in Cognitive Radio Network

**DOI:** 10.3390/s20051264

**Published:** 2020-02-26

**Authors:** Yuan Fang, Lixiang Li, Yixiao Li, Haipeng Peng, Yixian Yang

**Affiliations:** 1 Information Security Center, State Key Laboratory of Networking and Switching Technology, Beijing University of Posts and Telecommunications, Beijing 100876, China; fangyuan@bupt.edu.cn (Y.F.); doveliyixiao@163.com (Y.L.); penghaipeng@bupt.edu.cn (H.P.); yxyang@bupt.edu.cn (Y.Y.); 2 School of Computer Science and Technology, Henan Polytechnic University, 2001 Century Avenue, Jiaozuo 454003, China; 3 Guizhou University, Guizhou Provincial Key Laboratory of Public Big Data, Guiyang 550025, China

**Keywords:** compressed sensing, wideband spectrum sensing, sub-Nyquist sampling, cognitive radio network

## Abstract

For wireless communication networks, cognitive radio (CR) can be used to obtain the available spectrum, and wideband compressed sensing plays a vital role in cognitive radio networks (CRNs). Using compressed sensing (CS), sampling and compression of the spectrum signal can be simultaneously achieved, and the original signal can be accurately recovered from the sampling data under sub-Nyquist rate. Using a set of wideband random filters to measure the channel energy, only the recovery of the channel energy is necessary, rather than that of all the original channel signals. Based on the semi-tensor product, this paper proposes a new model to achieve the energy compression and reconstruction of spectral signals, called semi-tensor product compressed spectrum sensing (STP-CSS), which is a generalization of traditional spectrum sensing. The experimental results show that STP-CSS can flexibly generate a low-dimensional sensing matrix for energy compression and parallel reconstruction of the signal. Compared with the existing methods, STP-CSS is proved to effectively reduce the calculation complexity of sensor nodes. Hence, the proposed model markedly improves the spectrum sensing speed of network nodes and saves storage space and energy consumption.

## 1. Introduction

**Figure 1:**
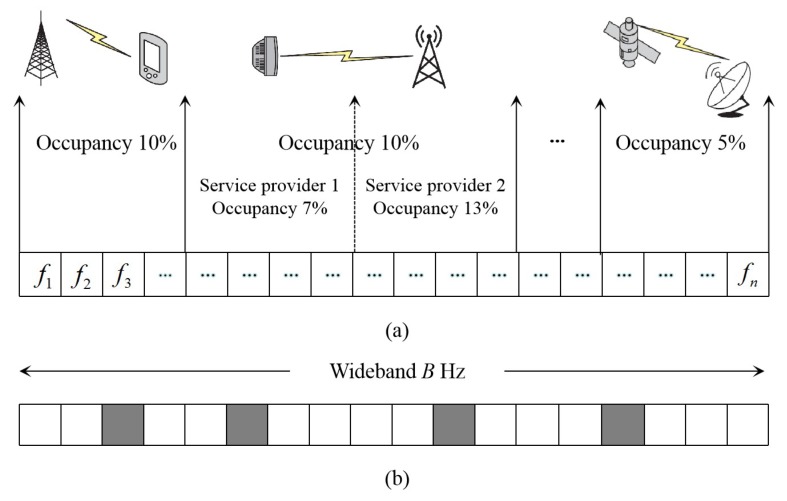
(**a**) Spectrum occupancy in a real scenario. (**b**) The grey areas indicate the occupied channels and the white areas indicate the idle channels.

The number of wireless users and the service quality requirements are increasing with the rapid development of wireless communication technology. A large number of users are demanding high-speed and high-bandwidth communication services, so the limited spectrum resource has become unusually valuable. Almost all the governments now use the principle of static allocation to authorize the fixed spectrum for different services users. However, the investigation report released by the Federal Communications Commission (FCC) stated that the use rate of the licensed spectrum is low, for the authorized users are idle on most occasions [1,2]. As a result, many spectrum holes exist in the daily use of the spectrum [3], as shown in Figure 1, which shows the wideband spectrum occupancy in real life scenarios. In this case, Mitola et al. proposed cognitive radio (CR), which became a suitable solution to the low spectrum use and the lack of spectrum resources [4]. The technical idea of CR is that a secondary user (SU) can dynamically look for opportunities to access the licensed spectrum without interfering with the primary user (PU). Hence, the spectrum holes in real scenes can be fully used. Chiwewe et al. summarized the CR methods related to industrial applications [5], including CR architecture, spectrum sensing, dynamic spectrum access (DSA), and interference management.

**Figure 2:**
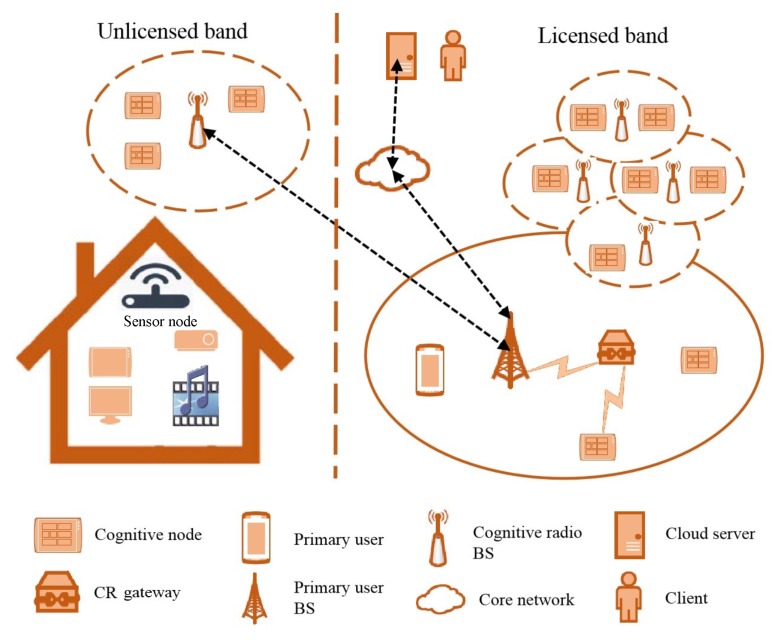
Communication architecture for a cognitive radio network.

Recently, the cognitive radio network (CRN) has been widely used in various wireless communication fields, such as ad-hoc networks, Internet of things (IoT) networks, and fifth generation wireless systems (5G) [6,7,8]. The cognitive characteristics of a CRN are used to distinguish the state of the network and make decisions and responses in daily life. The cognitive radio network has recently drawn widespread attention as it can provide flexible wideband services and stable operation in dynamic spectrum scenarios. The cognitive radio network can access the licensed spectrum of wireless communication and detect the spectrum usage status of the primary user through spectrum sensing [9,10,11]. For example, Figure 2 depicts a cognitive radio network structure in a wireless communication environment. However, with the rapidly growing demand for large-scale sensor deployments and mass data services, the lack of spectrum resources limits the development of the IoT [12]. The main challenge of the cognitive radio network is to provide sufficient available wideband spectrum resources for a great number of SU sensor nodes. Therefore, spectrum sensing and the sharing of dynamic spectrum have become quite important.

Spectrum sensing is the most critical and basic process in cognitive networks. The main purpose of spectrum sensing is to find idle spectrum through fast, efficient, and reliable methods, which allows the CR to achieve dynamic spectrum access. Spectrum sensing is further divided into narrowband spectrum sensing (NSS) and wideband spectrum sensing (WSS). The traditional narrowband spectrum sensing algorithms include matched filtering, energy detection, and feature extraction [13,14]. To simultaneously sense a plurality of channels, wideband spectrum sensing is an effective solution in CR. The CR network should handle a wide range of broadband spectra, i.e., from hundreds of MHZ to a few GHZ. One of traditional wideband spectrum sensing approaches is to use an analog-to-digital converter (ADC) to sample a wideband signal at an Nyquist rate, but large amounts of sampling are required. To reduce the amount of data perception and transmission in cognitive wireless networks, Tian et al. applied compressed sensing technology [15,16] to broadband spectrum sensing, called compressed spectrum sensing (CSS) [17]. CSS can reconstruct the spectral edge information directly from the measurement by taking advantage of the sparseness of the spectral signal and the detection technique of the wavelet-based edge. In addition, to achieve anti-noise robustness, Tian et al. also studied the wideband spectrum compression sensing algorithm based on cyclic feature detection [18]. Based on compressed sensing, Kirolos et al. created an analog-to-information converter (AIC) in which the analog signal can be directly sampled at a sub-Nyquist rate by random demodulation [19]. Mishali et al. modified the AIC model and proposed a modulation wideband converter (MWC) model for multiple sampling channels [20], and their model was found to be highly robust in noisy environments.

Although the above methods can achieve sub-Nyquist sampling to detect the spectrum channel occupancy state, the entire original signal spectrum or the whole power density spectrum must still be reconstructed. Havary et al. used a small number of filters to obtain a linear measurement of spectrum signal energy and directly reconstruct the signal energy [21]. In this way, the reconstruction of the entire spectrum is unnecessary. Once the energy of each channel is reconstructed, the dimension of the sparse energy vector is much lower than the spectral signal dimension. Due to the non-coherent property of energy detection, the priori information of a signal does not need to be known. However, these wideband compressed spectrum sensing algorithms still require a large number of wideband filters for perceptual calculation and storage [22,23,24]. Both the configuration complexity and the delay are high, which fail to meet the requirements of fast and accurate detection for wideband signals.

To solve these problems related to spectrum sensing, we propose a high-efficiency energy reconstruction compressed spectrum sensing algorithm based on the semi-tensor product in a cognitive radio network. In the proposed scheme, the wideband signal is sampled at a sub-Nyquist rate, and the prior information of the spectral signal is unneeded. The size of the measurement matrix of the CR node is much lower than that of the existing compressed spectrum sensing algorithms. The reconstruction process can be performed in parallel to reduce the delay. The main contributions of this paper are summarized as follows:

1. No relay on prior information. The prior information of the spectrum signal does not need to be known in advance, and we only reconfigure the channel energy. Using the sparsity of the channel energy, a wideband filter bank is used to obtain a linear measurement of the channel energy, whose value is used to determine whether the channel is occupied. Compared with the previous algorithms, the proposed algorithm focuses on the direct recovery of the spectrum channel energy instead of the restoring of the entire spectrum signal. Hence, the data processing operation is lower during the sensing process.
2. Low storage overhead and low sampling rate. Based on the compressed sensing theory, our algorithm can compress high-dimensional signals using low-dimensional sensing matrices. According to the characteristics of the semi-tensor product, fewer wideband filters for the proposed algorithm are required than traditional compressed spectrum sensing algorithms. Hence, the storage overhead of the CR node will be greatly reduced and the sensing process is more efficient.
3. Parallel reconstruction and less time required. The reconstruction method of semi-tensor compressed spectrum sensing can be implemented in parallel. Based on the characteristics of the semi-tensor product, the channel energy is simultaneously performed in multiple compressed sensing (CS) decoders so that the total spectrum sensing time is markedly reduced, which provides an important contribution to the immediacy of dynamic spectrum access.

## 2. Fundamental Knowledge

### 2.1. Cognitive Radio

The main idea of the cognitive radio (CR) system is to maximize the spectrum use by using idle bands [25,26]. Usually, most of the channels in the spectrum are free and they can be used by the second users (SUs). To find these spectrum holes without interfering with the primary user (PU), the CR system should use spectrum sensing to perceive and detect the spectrum.

Spectral detection is the judgment of the existence of a signal on the detected spectrum wideband, which is usually regarded as the following binary hypothesis problem:

(1) $$\left\{\begin{array}{c} H_{0}:x\left(t\right)=n\left(t\right)\\ H_{1}:x\left(t\right)=h\left(t\right)*s\left(t\right)+n\left(t\right) \end{array}\right.$$

where $H_{1}$ and $H_{0}$ indicate two states where the primary user exists or not, respectively, in the frequency band; x(t) represents the signal sequences received by the second user; s(t) is the signal sequences sent by the primary user,  h(t) is the channel gain; and n(t) is Gaussian white noise, which satisfies $n\left(t\right)∼N\left(0,\sigma^{2}\right)$. “*” is a time domain convolution operation.

Different detection methods can be used to construct the decision statistic V(x) by the obtained signal *x*, and comparing V(x) with the threshold value λ:

(2) $$V{\left(x\right)}_{\underset{D_{0}}{<}}^{\overset{D_{1}}{>}}\lambda ,\;or\left\{\begin{array}{c} V<\lambda ,D_{0}\\ V\geq \lambda ,D_{1} \end{array}\right.$$

where $D_{0}$ indicates the channel state is idle and $D_{1}$ indicates the channel state is occupied.

The perceived results are evaluated by two performance indicators:

(*i*) Detection probability:

(3) $$P_{d}=P\left(D_{1}/H_{1}\right)=P\left(Y\geq \lambda |H_{1}\right)$$

(ii) False alarm probability:

(4) $$P_{f}=P\left(D_{1}/H_{0}\right)=P\left(Y\geq \lambda |H_{0}\right)$$

The detection probability represents the possibility that the detection result is $D_{1}$ when the spectrum is occupied by the primary user in reality. The false alarm probability indicates that when the spectrum is not actually occupied by the primary user, the detection result is still $D_{1}$. The higher the detection probability, the lower the false alarm rate, and the more reliable the perception result.

### 2.2. Compressed Sensing

Suppose that the signal $x={\left[x_{1},x_{2},...,x_{N}\right]}^{T}$ with length *N* is compressible or *K*-sparse, and the signal is observed after a dimensionality reduction through the measurement matrix. Then, the available measurement vector *y* is given as follows:

(5) y=Φx

where Φ is the sensing matrix with size M×N(M≤N) and  *y* is the linear projection of the signal *x*. Since *x* is an *N*-dimensional signal and *y* is an *M*-dimensional signal, it is theoretically impossible to solve *x* while M≤N. Candes et al. demonstrated that if the sensing matrix satisfies the restricted isometry property (RIP), then the original signal can be reconstructed with high precision [27].

Candes et al. proved that as long as the measurement matrix Φ satisfies the RIP and $M\geq O(K\log_{2}N)$, the signal *x* can be accurately recovered with a probability approaching 1, which is marked as ${∥s∥}_{0}=K$ [16]. Currently, many famous matrices, like Gaussian random matrices and Bernoulli matrices, can satisfy RIP conditions, which are commonly used in compressed sensing applications [28,29].

For the purpose of recovering the original signal from the compressed sampling sequence, only the transformation before the reverse solution is needed. The solution formula can be solved by locally optimal greedy iterative algorithms, such as matching pursuit (MP) algorithm, orthogonal matching pursuit (OMP) algorithm, and the regular orthogonal matching pursuit (ROMP) algorithm, etc. [30,31,32].

### 2.3. Compressed Spectrum Sensing

It is assumed that the *W* Hz spectrum is used by the primary and secondary users, or it is shared by the nodes of an ad-hoc network, or the secondary network of the cognitive radio attempts to access the licensed spectrum for the secondary communication. If the communication of each node in the network requires *B* Hz, then N=W/B Hz is defined as the number of all channels, and $f_{i}$ is used to indicate the center frequency of the *i*th channel. Each node receives the entire spectrum using a wideband antenna, x(t), which is provided by each node is a broadband time domain signal.

Each node generates and stores a random matrix Φ with size K×N in advance, which is determined by the channel occupancy sparsity. These matrices are much smaller than the total channel number *N*. Each node uses this matrix to generate *K* wideband filters ${\left\{\left.H_{k}\left(f_{i}\right)\right\}\right.}_{k=1}^{K}$. The transformation function of the *k*th filter is given as follows [17]:

(6) $$H_{k}\left(f_{i}\right)={\left[\Phi \right]}_{ki},\;k=1,2,\cdots ,K,\;i=1,2,\cdots ,N.$$

It can be assumed that the input wideband signal x(t) of the node is transformed to obtain the time series vector, then the node sends the wideband signal to the filter, and the output of the *k*th filter is:

(7) $$z_{k}=Conv\left(x_{t},h_{k}\right)$$

where conv(·,·) represents a convolution operation and $h_{k}$ is an impulse response sequence. A K×1-dimensional energy vector *y* can be obtained from Equation (7)

(8) $$\begin{array}{c} y_{k}={z_{k}}^{H}z_{k},\;k=1,2,\cdots ,K\\ y={\left[y_{1},y_{2},\cdots ,y_{k}\right]}^{T} \end{array}$$

where ${\left(\cdot \right)}^{T}$ and ${\left(\cdot \right)}^{H}$ represent the transpose transformation and the conjugate transpose transformation of a matrix, respectively; $E_{i}$ is used to represent the signal energy received by the *i*th channel. Mathematically, we have:

(9) $$E_{i}=\int_{f_{i}-B/2}^{f_{i}+B/2}Fx(t)df$$

where F represents the continuous Fourier transform of a signal. Assume that the frequency response of each filter is approximately constant, and the equation for the *i*th channel of the *k*th filter is $H_{k}\left(f_{i}\right)=\Phi_{ki}$. So, the energy of the output signal *k*th filter can be written as:

(10) $$y_{k}=\sum_{i=1}^{N}{\left|\left.H_{k}\left(f_{i}\right)\right|\right.}^{2}E_{i},\;k=1,2,\cdots ,K.$$

Equation (10) can be vectorized as:

(11) $$y=\bar{\Phi }e$$

where:

(12) $$\bar{\Phi }=\left[\begin{array}{cccc} {\left|H_{1}\left(f_{1}\right)\right|}^{2} & {\left|H_{1}\left(f_{2}\right)\right|}^{2} & \cdots & {\left|H_{1}\left(f_{N}\right)\right|}^{2}\\ {\left|H_{2}\left(f_{1}\right)\right|}^{2} & {\left|H_{2}\left(f_{2}\right)\right|}^{2} & \cdots & {\left|H_{2}\left(f_{N}\right)\right|}^{2}\\ \vdots & \vdots & \ddots & \vdots \\ {\left|H_{K}\left(f_{1}\right)\right|}^{2} & {\left|H_{K}\left(f_{2}\right)\right|}^{2} & \cdots & {\left|H_{K}\left(f_{N}\right)\right|}^{2} \end{array}\right]$$

In this paper, $\bar{\Phi }$ is a matrix whose elements are the square of the absolute value of the elements in the random matrix Φ, and $e={\left[E_{1},E_{2},\ldots ,E_{N}\right]}^{T}$ is the received energy vector of different channels. Our goal is to recover the vector *e* with length *N* using the measurement vector *y* with length *K*.

### 2.4. Semi-Tensor Product

Cheng et al. proposed the concept of the semi-tensor product (STP) of the matrix, is was a generalization of the traditional matrix product [33]. The multiplication of two matrices can be accomplished using STP when the two matrices do not satisfy the dimension matching condition of the traditional matrix multiplication. STP can be used in many fields, such as image encryption, game theory, and body-to-body networks [34,35]. Suppose *X* is a row vector with dimension np and *Y* is a column vector with dimension *p*. Then, we split *X* into *p* equal-sized blocks $X^{1},X^{2},\cdots ,X^{p}$, and each of these blocks has 1×n lines. The semi-tensor product is represented by ⋉, and it is defined as:

(13) $$\left\{\begin{array}{c} X⋉Y=\sum_{i=1}^{p}X^{i}y_{i}\in R^{n},\\ Y^{T}⋉X^{T}=\sum_{i=1}^{p}y_{i}{\left(X^{i}\right)}^{T}\in R^{n}. \end{array}\right.$$

For $A\in M_{m\times n},B\in M_{p\times q}$, if *n* is a factor of *p*, there is nh=p, and it is recorded as A<hB. Otherwise, if *p* is a factor of *n*, there is n=ph, and it is recorded as A>hB. The left semi-tensor product of *A* and *B* is defined as C=A⋉B. *C* is composed of m×p blocks, and each block is:

(14) $$C^{ij}=A^{i}⋉B_{j}$$

where $A^{i}$ is the *i*th row of *A*, $B_{j}$ is the *j*th column of *B*, i=1,2,⋯m, and j=1,2,⋯,q. The multiplication of the semi-tensor product can also be defined by the Kronecker product. The Kronecker product of two matrices $A\in M_{m\times n},B\in M_{p\times q}$ is defined as:

(15) $$A⊗B=\left[\begin{array}{ccc} a_{11}B & \cdots & a_{1n}B\\ \vdots & \ddots & \vdots \\ a_{m1}B & \cdots & a_{mn}B \end{array}\right]\in R^{mp\times nq}$$

According to [23], the semi-tensor product of $A\in M_{m\times n},B\in M_{p\times q}$ is A⋉B=(A⊗Ittnn)⋉(B⊗Ittpp), where *t* is the least common multiple of *n* and *p*. There are (A⊗Ittnn)∈Rmtmtn×tn×t and (B⊗Ittpp)∈Rt×qtt×qtpp so that A⋉B∈Rmtmtn×qtqtppn×qtqtpp. When p=n, there is $A⋉B=\left(A⊗I_{1}\right)\left(B⊗I_{1}\right)=AB$, and the semi-tensor product degenerates into standard matrix multiplication.

## 3. Improved Compressed Spectrum Sensing

**Figure 3:**
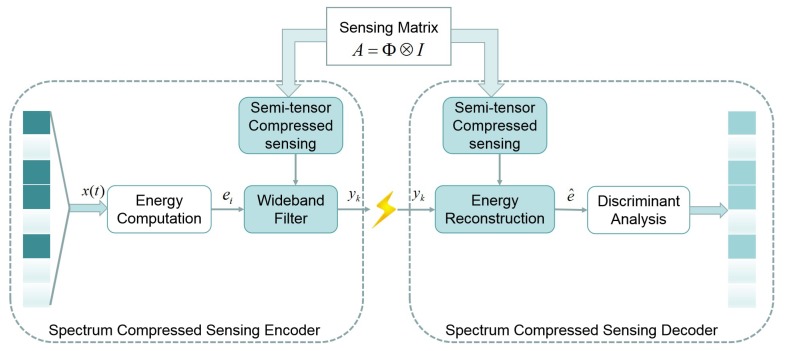
A scheme of semi-tensor product compressed spectrum sensing (STP-CSS). The channel energy is calculated and compressed in the encoder and reconstructed in the decoder to determine the occupancy of the spectrum channel.

In this paper, we consider the CR network scenario where many channels are free holes. To exploit the holes and avoid any interference caused to PUs, the system has to sense the spectrum and detect it before the transmission of SU signals. The algorithm structure of the proposed scheme is shown in Figure 3, which consists of the STP-CSS encoder and decoder. We can calculate the channel energy from the received spectrum signal and compress it in the encoder on sensors of SUs, and then reconstruct the channel energy in the decoder terminal to determine the occupancy state of the spectrum channel after the transmission.

Assume that in a wideband wireless network, the entire spectrum bandwidth to be perceived is supposed to be *W* HZ, which is in the frequency range $\left[f_{0},f_{N}\right]$. Then, it is divided into *N* channels with bandwidth B=W/N Hz, and $f_{i}$ represents the center frequency of the *i*th channel. Each SU monitors a time domain wideband signal x(t). An additive white Gaussian noise (AWGN) with mean 0 and variance $\sigma^{2}$ is distributed to the signal.

Havary et al. proposed the compressed spectrum sensing algorithm [21]. In their algorithm, each SU node should independently generate and store *M* wideband filters (M≤N) to perform the compressed sensing on the received signal energy. In this case, we select a common factor *P* of *M* and *N*, and each node only generates and stores an M/P×N/P-dimensional Gaussian random matrix ϕ. The matrix ϕ with size M/P×N/P is convolved with a *P*-dimensional unit matrix, resulting in a matrix *A* with dimension M×N. The wideband signal x(t) is then fed into the filter that is generated by this matrix *A* via discrete Fourier transformation (DFT) for the parallel processing. The energy of the *i*th channel is calculated according to the received signal x(t) by the following equation:

(16) $$e_{i}=\int_{f_{i}-\frac{B}{2}}^{f_{i}+\frac{B}{2}}{\left|Fx(t)\right|}^{2}df\begin{array}{cc} ,\; & i=1,2,\cdots ,N. \end{array}$$

The channel energy vector is $E\in R^{N}$.The conversion equation of the *i*th channel and the *k*th filter is:

(17) $$\begin{array}{cc} A_{k,i}={\left(\varphi_{\frac{M}{P},\frac{N}{P}}⊗I_{p}\right)}_{k,i}, & \begin{array}{cc} k=1,2,\cdots ,M, & i=1,2,\cdots ,N. \end{array} \end{array}$$

Then, we have:

(18) $$A=\Phi ⊗I=\left(\begin{array}{cccccccccc} a_{11} & \cdots & 0 & a_{12} & \cdots & 0 & \begin{array}{c} \cdots \end{array} & a_{1\frac{N}{P}} & \cdots & 0\\ \vdots & \ddots & \vdots & \vdots & \ddots & \vdots & \begin{array}{c} \cdots \end{array} & \vdots & \ddots & \vdots \\ 0 & \cdots & a_{11} & 0 & \cdots & a_{12} & \begin{array}{c} \cdots \end{array} & 0 & \cdots & a_{1\frac{N}{P}}\\ a_{21} & \cdots & 0 & a_{22} & \cdots & 0 & \begin{array}{c} \cdots \end{array} & a_{2\frac{N}{P}} & \cdots & 0\\ \vdots & \ddots & \vdots & \vdots & \ddots & \vdots & \begin{array}{c} \cdots \end{array} & \vdots & \ddots & \vdots \\ 0 & \cdots & a_{21} & 0 & \cdots & a_{22} & \begin{array}{c} \cdots \end{array} & 0 & \cdots & a_{2\frac{N}{P}}\\ \vdots & \vdots & \vdots & \vdots & \vdots & \vdots & \begin{array}{c} \ddots \end{array} & \vdots & \vdots & \vdots \\ a_{\frac{M}{P}1} & \cdots & 0 & a_{\frac{M}{P}2} & \cdots & 0 & \begin{array}{c} \cdots \end{array} & a_{\frac{M}{P}\frac{N}{P}} & \cdots & 0\\ \vdots & \ddots & \vdots & \vdots & \ddots & \vdots & \begin{array}{c} \cdots \end{array} & \vdots & \ddots & \vdots \\ 0 & \cdots & a_{\frac{M}{P}1} & 0 & \cdots & a_{\frac{M}{P}2} & \begin{array}{c} \cdots \end{array} & 0 & \cdots & a_{\frac{M}{P}\frac{N}{P}} \end{array}\cdots \right)$$

The size of the matrix *A* is M×N, the size of ϕ is $\frac{M}{P}\times \frac{N}{P}$, and *I* is the unit matrix with dimension *P*.

The output of the filter is:

(19) $$y=\Phi ⋉E=(\Phi ⊗I_{P})E=AE.$$

The signal energy generated from the *k*th filter is:

(20) $$y_{k}=\sum_{i=1}^{N}A_{k,i}E_{i}.$$

(*i*) First we calculate the front *P* row element of *Y*, and we obtain:

(21) $$\begin{array}{c} y_{1}=a_{11}e_{1}+a_{12}e_{P+1}+...+a_{1\frac{N}{P}}e_{(\frac{N}{P}-1)P+1}=\sum_{j=1}^{\frac{N}{P}}a_{1j}e_{(j-1)P+1}\\ y_{2}=a_{11}e_{2}+a_{12}e_{P+2}+...+a_{1\frac{N}{P}}e_{(\frac{N}{P}-1)P+2}=\sum_{j=1}^{\frac{N}{P}}a_{1j}e_{(j-1)P+2}\\ \begin{array}{cccccccccc} & \vdots & & & & & & & & \end{array}\\ y_{P}=a_{11}e_{i}+a_{12}e_{2P}+...+a_{1\frac{N}{P}}e_{N}=\sum_{j=1}^{\frac{N}{P}}a_{1j}e_{jP} \end{array}$$

That is, for i=1,2,⋯,P, we have:

(22) $$\begin{array}{c} y_{i}=a_{11}e_{i}+a_{12}e_{P+i}+...+a_{1\frac{N}{P}}e_{(\frac{N}{P}-1)P+i}\\ \begin{array}{cc} & =\sum_{j=1}^{\frac{N}{P}}a_{1j}e_{(j-1)P+i} \end{array}\\ \begin{array}{cc} & = \end{array}\left(a_{11},a_{12},\cdots ,a_{1\frac{N}{P}}\right)\cdot {\left(e_{i},e_{P+i},\cdots e_{(\frac{N}{P}-1)P+i}\right)}^{T} \end{array}$$

(ii) Continue to calculate the elements from P+1 to *M* lines of *Y*, then we get

(23) $$\begin{array}{c} y_{P+i}=a_{21}e_{i}+a_{22}e_{P+i}+\cdots +a_{2\frac{N}{P}}e_{(\frac{N}{P}-1)P+i}\\ \begin{array}{cc} & =\sum_{j=1}^{\frac{N}{P}}a_{2j}e_{(j-1)P+i} \end{array}\\ \begin{array}{cc} & = \end{array}\left(a_{21},a_{22},\cdots ,a_{2\frac{N}{P}}\right)\cdot {\left(e_{i},e_{P+i},\cdots ,e_{(\frac{N}{P}-1)P+i}\right)}^{T}\\ \begin{array}{c} \begin{array}{cccc} \begin{array}{cccccccccc} & \vdots & & & & & & & & \end{array} & & & \end{array}\\ \begin{array}{c} y_{(\frac{M}{P}-1)P+i}=a_{\frac{M}{P}1}e_{i}+a_{\frac{M}{P}2}e_{P+i}+\cdots +a_{\frac{M}{P}\frac{N}{P}}e_{(\frac{N}{P}-1)P+i}\\ \begin{array}{cc} & =\sum_{j=1}^{\frac{N}{P}}a_{\frac{M}{P}j}e_{(j-1)P+i} \end{array}\\ \begin{array}{cc} & = \end{array}\left(a_{\frac{M}{P}1},a_{\frac{M}{P}2},\cdots ,a_{\frac{M}{P}\frac{N}{P}}\right)\cdot {\left(e_{i},e_{P+i},\cdots ,e_{(\frac{N}{P}-1)P+i}\right)}^{T} \end{array} \end{array} \end{array}$$

According to Equations (20)–(23), the process of the spectral channel energy passing through the wideband filter, that is, the compressed sensing process, can be expressed as:

(24) $$\begin{array}{c} \left(\begin{array}{c} y_{i}\\ y_{P+i}\\ \vdots \\ y_{(\frac{M}{P}-1)P+i} \end{array}\right)=\left(\begin{array}{cccc} a_{11} & a_{12} & \cdots & a_{1\frac{N}{P}}\\ a_{21} & a_{22} & \cdots & a_{2\frac{N}{P}}\\ \vdots & \vdots & \ddots & \vdots \\ a_{\frac{M}{P}1} & a_{\frac{M}{P}2} & \cdots & a_{\frac{M}{P}\frac{N}{P}} \end{array}\right)\left(\begin{array}{c} e_{i}\\ e_{P+i}\\ \vdots \\ e_{(\frac{N}{P}-1)P+i} \end{array}\right) \end{array}$$

Thus, the final reconstructed channel energy can be defined as:

(25) $$\hat{E}={\left[e^{1},e^{2},\cdots ,e^{P}\right]}^{T}$$

where $e^{i}={\left(e_{i},e_{P+i},\cdots ,e_{(\frac{N}{P}-1)P+i}\right)}^{T},y^{i}={\left(y_{i},y_{P+i},\cdots ,y_{(\frac{M}{P}-1)P+i}\right)}^{T}$.

We can use the traditional compressed sensing reconstruction algorithms in our model because $\Phi ⋉E=(\Phi ⊗I_{P})E$. According to the structure $\Phi ⊗I_{P}$, the instance reconstruction in the STP-CSS model can be converted into *P* independent reconstruction processes, and then parallel reconstruction processing uses the orthogonal matching pursuit algorithm in the software aspect. The spectrum sensing reconstruction algorithm based on channel energy reconstruction is shown in Algorithm 1. According to Equation (25), the decoder terminal should have *p* servers to meet the parallel reconstruction requirement in terms of hardware aspect.
**Algorithm 1** Parallel reconstruction algorithm of the proposed STP-CSS Model.**Input:** The received signal $y\in R^{N}$ and measurement matrix $A\in R^{\frac{M}{P}\times \frac{N}{P}}$
**Output:** The reconstructed channel energy *e* **for**i=1 to *p* **do**    $y^{i}={\left(y_{i},y_{P+i},...,y_{(\frac{M}{P}-1)P+i}\right)}^{T}\in R^{\frac{M}{P}}$**end for****for**i=1 to *p* **do**    $e^{i}\to \begin{array}{ccc} CS & reconstruction & algorithm \end{array}\to \left(y^{i},A\right)$**end for****return**$e:=vec(X^{T}).$ vec(*E*) denotes the vectorization of *E*


For purpose of determining whether each channel is occupied or idle, the energy detection result obtained from Algorithm 1 is compared with the threshold λ:

(26) $$\left\{\begin{array}{c} E_{i}<\lambda \begin{array}{cc} , & H_{0} \end{array}\\ E_{i}>\lambda \begin{array}{cc} , & H_{1} \end{array} \end{array}\right.$$

## 4. Experimental Results

**Figure 4:**
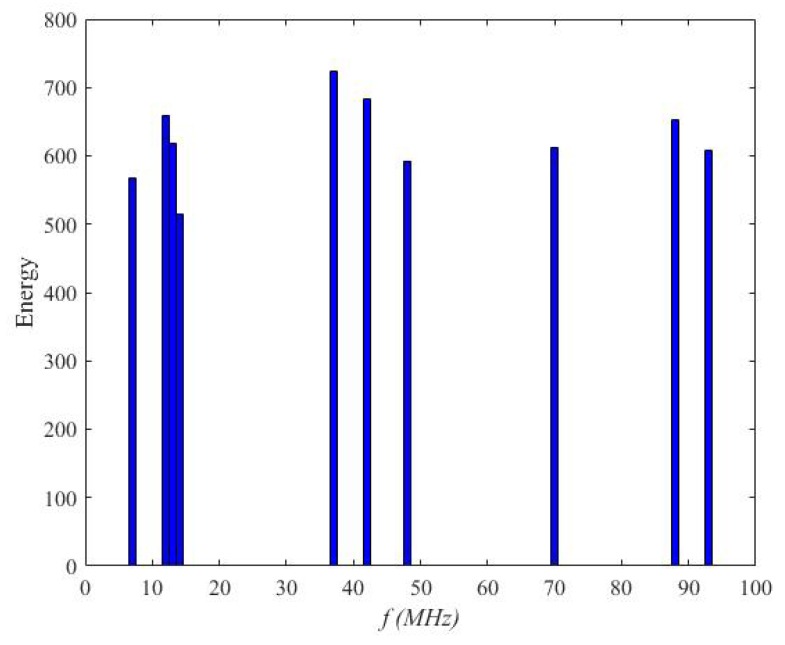
An example of the distribution of the channel energy. The entire spectrum bandwidth to be perceived was 100 HZ, which is in the frequency range of (0; 100) MHz. The whole spectrum was divided into 100 channels.

In the experiment, we used the following broadband time domain signal model:

(27) $$x\left(t\right)=\sum_{i=1}^{N}\sqrt{P_{i}B_{i}}\cos\left(2\pi f_{i}\left(t-\alpha \right)\right)+n\left(t\right)$$

where $P_{i}$ and $B_{i}$ represent the received power and the channel width, respectively; $f_{i}$ denotes the center frequency of the *i*th channel at the SU; α is a random time offset, and $n\left(t\right)∼N\left(0,\sigma^{2}\right)$. In this experiment, we perceived the spectrum frequency range from 100 to 200 MHz, so the total band width W=100 MHz, which was divided into N=100 communication channels with the same band width $B_{i}=1$ MHz. Figure 4 depicts an example of the occupancy of channel energy at a communication moment. In Figure 4, the signal channel had a signal of $f_{i}=\left(7,12,13,14,15,37,42,48,69,88,93\right)$ MHz. Table 1 lists the values of the other parameters used in the proposed algorithm. We used MATLAB software (MathWorks, USA) to simulate our algorithm.

### 4.1. Detection Rates

**Figure 5:**
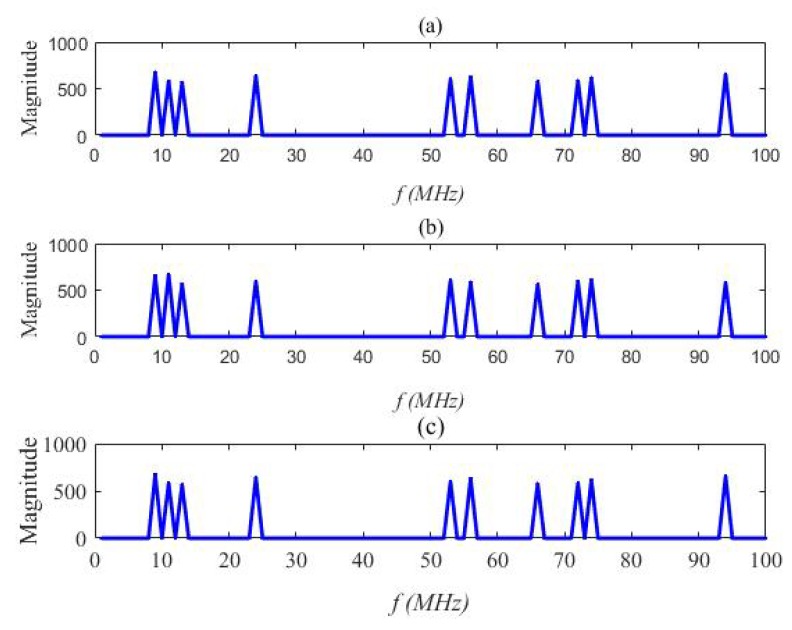
Comparison of the original signal and the STP-CSS reconstructed signal when M=80. (**a**) The original energy; (**b**) the reconstructed energy of the compressed spectrum sensing algorithm; (**c**) the reconstructed energy of the semi-tensor compressed spectrum sensing algorithm.

**Figure 6:**
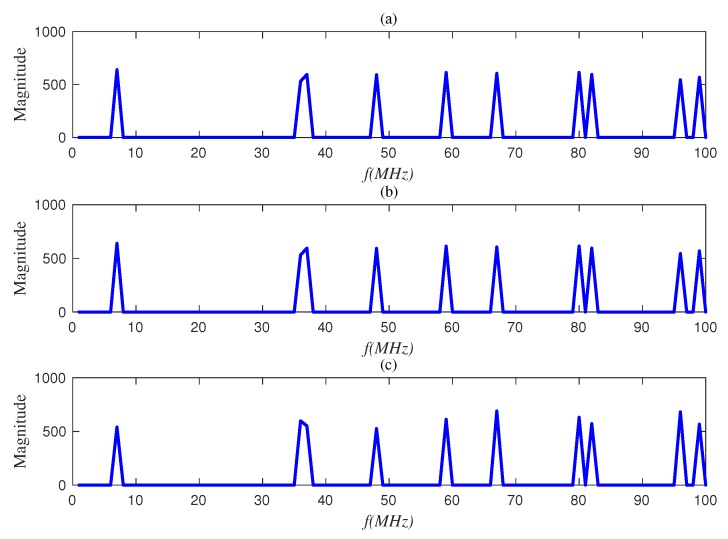
Comparison of the original signal and the STP-CSS reconstructed signal when M=100: (**a**) the original energy, (**b**) the reconstructed energy of the compressed spectrum sensing algorithm, and (**c**) the reconstructed energy of the semi-tensor compressed spectrum sensing algorithm.

**Figure 7:**
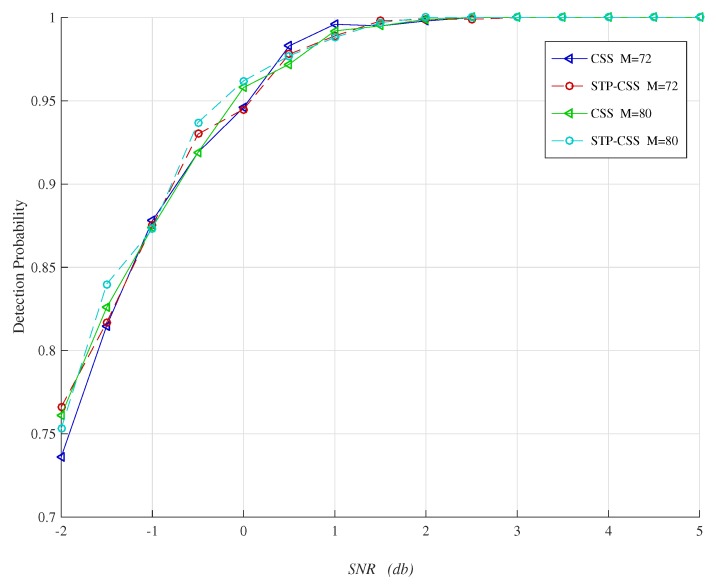
Comparison of the reconstructed signal in compressed spectrum sensing (CSS) and STP-CSS, where the numbers of the measurement matrix were set to 72 and 80.

**Figure 8:**
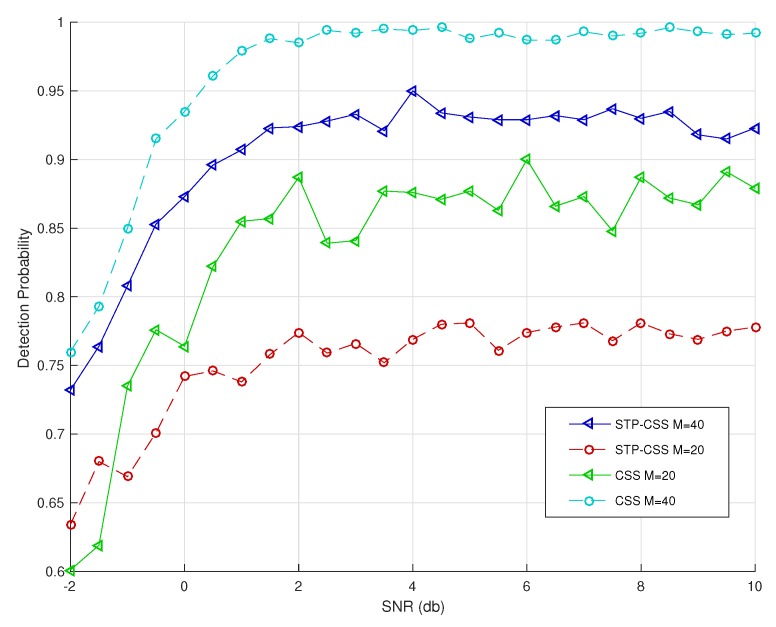
Comparison of the reconstructed signal in CSS and STP-CSS, where the numbers of the measurement matrix were set to 20 and 40.

We used a statistical simulation method to obtain the detection probability *P* corresponding to the false positive probability. Figure 5 and Figure 6 compare the reconstructed energies generated by the proposed semi-tensor product compressed spectrum sensing algorithm (STP-CSS) and the traditional compressed spectrum sensing algorithm (CSS) for channel energy recovery, where the numbers *M* of the filters were set to 80 and 100, respectively. The energy recovery effect of STP-CSS was similar to that of the CS algorithm. Figure 7 and Figure 8 compare the results of the detection probability between the proposed algorithm and the traditional compressed sensing algorithm for spectrum detection under different signal-to-noise ratio (SNR) conditions. In Figure 7, the number of detecting channels was set to be 100 and filters were set to be 80 and 72, respectively. In Figure 8, the number of detecting channels was set to be 100 and filters were set to be 40 and 20, respectively. We changed the SNR from −2 dB to 5 dB, and compared our algorithm with the traditional compressed sensing algorithm using a random matrix such as Gaussian Bernoulli and tent chaotic matrix as the measurement matrix. To be representative and authoritative, each experiment was conducted 100 times each under the same conditions, and the average detection rate was regarded as the experimental result. For each communication channel, the detection probability was calculated as follows:

(28) $$\begin{array}{c} Pd_{i}=\mathrm{Pr}ob\left\{E_{i}>\lambda |H_{1}\right\} \end{array}$$

where $H_{1}$ indicates that the real state of the channel is occupied. If the recovered channel energy $E_{i}$ was greater than the threshold λ and the true state of the channel was $H_{1}$, the channel was successfully detected. Figure 8 shows that the detection rates of the signal energy for the proposed method was similar to that of the traditional compressed spectrum sensing method when the compression ratio was low. As can be seen from Figure 7, the detection rates of the signal energy for the traditional compressed spectrum sensing method were similar to that of the proposed method when the compression ratio was high.

### 4.2. Energy Efficiency

The use of compressed sensing theory can reduce the amount of data transmitted, thereby reducing the communication energy consumption of sensor nodes. The energy consumption in CR networks is usually dominated by wireless communication. Thus, the energy model can be formulated as follows [36]:

(29) $$E_{n}=C_{e}\times M\times b$$

where $C_{e}$ indicates the energy consumption of transmitting 1 bit of data. The average energy consumption per bit in wireless communication is set to $C_{e}=3\;nJ/bit$. *M* is the amount of data to be transmitted, which can be formulated as M=m+p [37]; *b* is the bit resolution, and we set b=16 to satisfy its requirement. Using the number of sensing channels of 100, 200, 300, 400, 1000, 2000, 3000, and 4000, we calculated the energy consumption with different compression ratios when p=2, and the calculation results are shown in Table 2, where the energy was measured in $10^{3}$ nJ. When the compression ratio was 1, the algorithm degenerated to the traditional spectrum sensing without using compressed sensing. From Table 2, we find that a larger compression ratio led to lower energy consumption for transmitting the same-sized channel energy. For example, in Table 2, compared with the traditional spectrum sensing algorithm without CS theorem, the 0.4 compression ratio could save 2880, 5760, 28,800, and 57,600 mJ of energy for channels numbers of 100, 200, 1000, and 2000, respectively. In addition, the proposed algorithm only needed to transmit the channel energy and did not need to transmit the channel signal, which could also considerably reduce transmission and reconstruction energy consumption. Thus, the proposed model was energy-efficient, saving the energy resources of sensor nodes in CR networks.

### 4.3. Computation Complexity

Due to the semi-tensor product theory used in the proposed compressed spectrum sensing algorithm, the dimension of the measurement matrix *A* is m/p×n/p, instead of the dimension of the measurement matrix in the traditional CS, which is m×n, thereby reducing hardware overhead. Compared with m(n−1) times float number addition operations and mn times float number multiplication operations in traditional compressed spectrum sensing, the times float number addition operations in the proposed algorithm are m(n/p−1), and the times float number multiplication operations are m(n/p+1). The complexity of traditional spectrum compressed sensing is $O(n^{3})$, and the complexity of the proposed spectrum compressed sensing is O(n) [37]. In the experiment, the channel energy vector was $e\in R^{100\times 1}$. If *n* and *p* are set to 80 and 2, respectively, then $A⊗I\in R^{80\times 100}$, saving 80×50 float number addition operations and 80×49 float number multiplication operations. The results showed that a larger *p* could save more computing resources. The experiments showed that our proposed algorithm could effectively reduce the computational complexity of CR network sensing nodes.

### 4.4. Running Time

**Figure 9:**
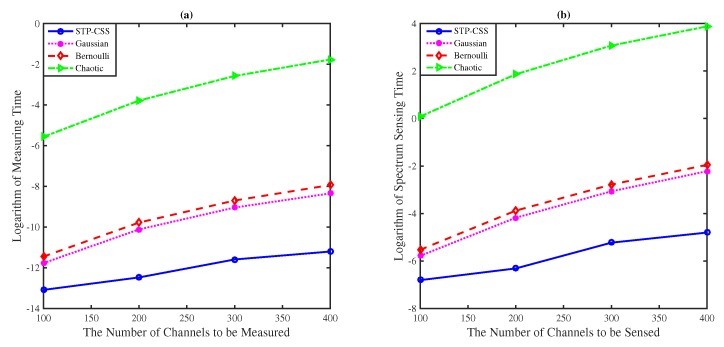
The comparisons of time costs on the measurement process and the whole spectrum sensing process. The numbers of the channels were set to 100, 200, 300, and 400: (**a**) The logarithm of the measurement time. (**b**) The logarithm of the spectrum sensing time.

Since the reconstruction process of the semi-tensor product compressed spectrum sensing model can be converted into *p* independent parallel reconstruction instances, considerable time is saved in the spectrum sensing process. In Figure 9, when the number of the channels was in the range [100,400], the proposed algorithm and the traditional compressed spectrum sensing algorithm, using a random matrix such as Gaussian Bernoulli and tent chaotic matrix as the measurement matrix, were applied for channel energy reconstruction. Figure 9a compares the time of the channel energy measurement process. Since the sensing nodes of spectrum sensing required extremely high measurement time, the proposed method provided a significant improvement. Figure 9b compares the time of the whole spectrum sensing process. Our algorithm provided significant time reductions.

**Figure 10:**
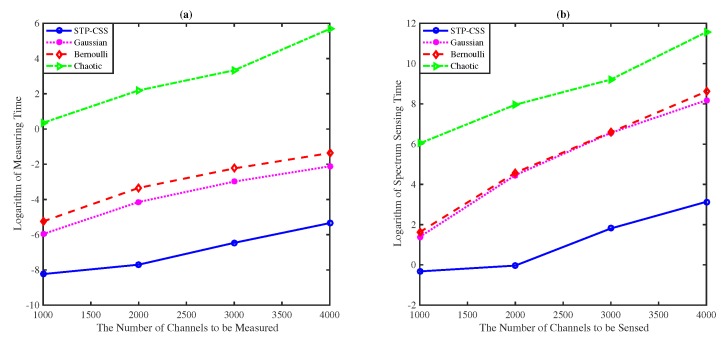
The comparisons of time costs on the measurement process and the whole spectrum sensing process. The numbers of the channels were set to 1000, 2000, 3000, and 4000. (**a**) The logarithm of measuring time. (**b**) The logarithm of sensing time.

Figure 10 compares the results between the measurement time of the channel energy and the time of the entire sensing process when the number of channels ranges from 1000 to 4000. In the proposed algorithm, the measurement matrix is composed of a small matrix and a *P*-dimensional unit matrix, so the sensing node and the data fusion center only should store the data of the original measurement matrix, and the reconstruction can be performed in parallel in the sensing process. Storage space and computing consumption can be reduced when the number of channels being perceived is extremely large.

### 4.5. Storage Space

**Figure 11:**
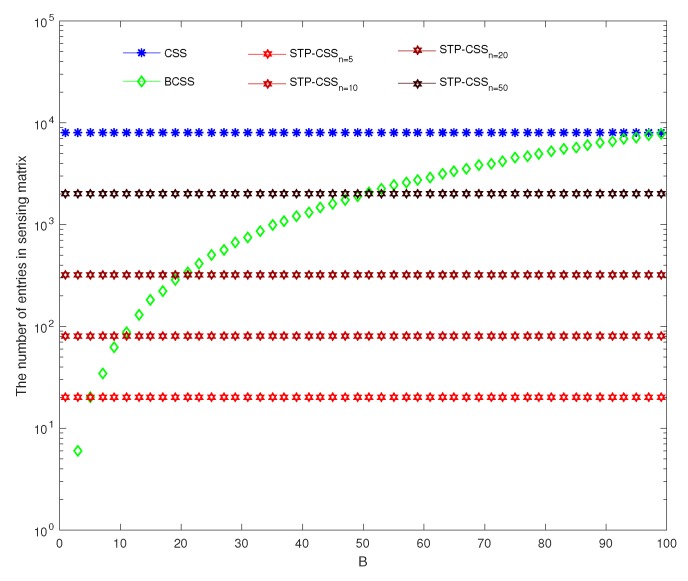
The comparison of storage performance in CSS, block-based compressed spectrum sensing (BCSS), and STP-CSS. The blue, green, and red lines represent CSS, BCSS, and STP-CSS, respectively. In the STP-CSS, the values of the columns *n* are set to 5, 10, 20, and 50.

**Figure 12:**
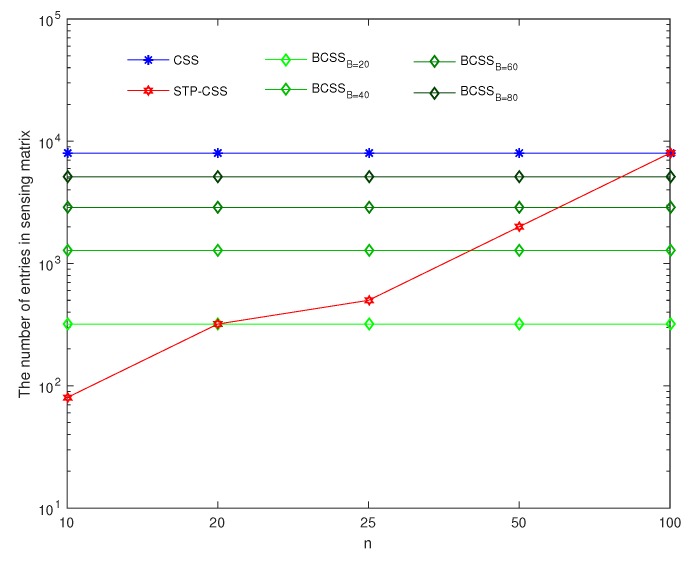
The comparison of storage performance in CSS, BCSS, and PTP-CSS. The blue, green, and red lines represent CSS, BCSS, and PTP-CSS, respectively. In the BCSS, the sizes of each block B are set to 20, 40, 60, and 80.

Gan et al. proposed a block-based signal compressed sensing model [38] called BCS, which efficiently reduces the storage space of measurement matrices in compressed spectrum sensing. The main idea of the block compressed spectrum sensing method is to first divide the spectrum signal into small blocks with the same size and then compress each block using the compressed sensing method with the same sensing matrix, and each block has the same size B×1. According to the block-based compressed spectrum sensing (BCSS) algorithm, the sensing matrix is a block diagonal matrix, which is given as follows:

(30) $$\Phi_{BCSS}=I_{N}⊗\Phi_{B}=\left(\begin{array}{cccc} \Phi_{B} & 0 & \cdots & 0\\ 0 & \Phi_{B} & \cdots & 0\\ \vdots & \vdots & \ddots & \vdots \\ 0 & \cdots & 0 & \Phi_{B} \end{array}\right)$$

where *N* is the number of the blocks in the signal. In the proposed STP-CSS model, for the same spectrum signal, the perceptual matrix can be described as:

(31) $$\Phi_{STP-CSS}=\Phi ⊗I_{p/n}=\left(\begin{array}{ccc} \varphi_{11}I_{p/n} & \cdots & \varphi_{1n}I_{p/n}\\ \vdots & \ddots & \vdots \\ \varphi_{m1}I_{p/n} & \cdots & \varphi_{mn}I_{p/n} \end{array}\right)$$

where $\varphi_{ij}$ is the element in the *i*th row and *j*th column of matrix Φ. Equations (30) and (31) show that the measurement matrix structures of BCSS and STP-CSS are similar. For the storage overhead, we compared the number of entries in the measurement matrices among CSS, BCSS, and STP-CSS. We found a more suitable method to minimize the storage space of the measurement matrix. We assume that *X* is a raw spectral signal with dimension p×1. The dimension of the measurement matrix in the conventional CSS is m×p. In BCSS, the signal is divided into *n* blocks with the same size B×1, and each block is compressed by a measurement matrix with dimension $\left\lfloor mB/p\right\rfloor B$. According to Equation (31), the dimension of the measurement matrix in STP-CSS is m·n/p×p·n/p, ie $mn^{2}/p$, where *n* is the factor of *p*. Therefore, the number of entries in the sensing matrix among CSS, BCSS, and STP-CSS are mp, $\left\lfloor mB/p\right\rfloor B$, and $mn^{2}/p$, respectively. Figure 11 and Figure 12 show the effect of *B* and *n* on the number of various measurement matrices, respectively. In Figure 11, we selected four different values of *n* in STP-CSS, i.e., n=5,10,20,50, satisfying *n* being the factor of *p*. In Figure 12, *B* was selected as different values for BCSS, i.e., B=20,40,60,80. From the simulation result, the perceived number of STP-CSS was smaller than that of the traditional CSS. In practical applications, since *B* in BCSS should be much larger than the sparse degree, it will not affect the recovery effect. Thus, *B* cannot be too small. Compared with BCSS, STP-CSS saved storage space when each block *B* was large enough. Overall, from the perspective of storage overhead, STP-CSS was more competitive than CSS and provided advantages over BCSS.

## 5. Conclusions

In this paper, we considered a realistic cognitive radio Internet of things (IoT) scenario where each communication channel is in neither in the occupied or idle state. We proposed a semi-tensor product compressed spectrum sensing method by using a set of wideband random filters. The basic strategy involves simultaneous detection of primary users on a set of wideband sub-bands, rather than only considering one single frequency band at a time. Obtaining channel energy measurements and determining the distribution of idle channels, we did not reconstruct the original signal, which greatly reduces the amount of data needed to be calculated. The biggest difference of the proposed algorithm from the existing algorithms is that semi-tensor product theory is implemented in the compressed spectrum sensing, which exponentially reduces the data storage space required in the CRN sensor node. The process of the perceptual reconstruction is performed in parallel, which improves the speed of spectrum sensing. Our scheme is useful for real-life scenarios where the spectrum situation changes dynamically. Simulation experiments showed that the detection rate of the compressed spectrum sensing algorithm using a semi-tensor product is not lower than that of the traditional algorithm, and the spectrum sensing time of the proposed scheme is significantly shorter.

## Figures and Tables

**Table 1:** Values of the experimental parameters.

Notation	Value	Description
$P_{i}$	0.02	Received power
$B_{i}$	1	Channel width
α	0.1	Time offset
σ	1	Variance of noise
λ	118	Judgment threshold

**Table 2:** Energy consumption ($10^{3}$ nJ) under different compression ratios.

Compression Ratio	*n* = 100	*n* = 200	*n* = 1000	*n* = 2000
1	4.896	9.696	48.096	96.096
0.8	3.936	7.776	38.496	76.896
0.72	3.552	7.008	34.656	69.12
0.4	2.016	3.936	19.296	38.496
0.2	1056	2.016	9.696	19.296

## Abbreviations

The following abbreviations are used in this manuscript:

CR	Cognitive radio
CRN	Cognitive radio network
CS	Compressed sensing
STP-CSS	Semi-tensor product compressed spectrum sensing
FCC	Federal Communications Commission
SU	Secondary user
PU	Primary user
DSA	Dynamic spectrum access
IoT	Cognitive radio network
5G	The fifth generation wireless systems
NSS	Narrowband spectrum sensing
WSS	Wideband spectrum sensing
ADC	Analog-to-digital converter
CSS	Compressed spectrum sensing
AIC	Analog-to-information converter
MWC	Modulation wideband converter
RIP	Restricted isometry property
OMP	orthogonal matching pursuit
MP	Matching pursuit
ROMP	regular orthogonal matching pursuit
STP	Semi-tensor product
AWGN	Additive white Gaussian noise
DFT	Discrete Fourier transformation
BCS	Block-based compressed sensing
